# A Computationally Efficient Filter for Reducing Shot Noise in Low S/N Data

**DOI:** 10.1371/journal.pone.0157595

**Published:** 2016-06-15

**Authors:** Mami Okada, Tomoe Ishikawa, Yuji Ikegaya

**Affiliations:** 1 Graduate School of Pharmaceutical Sciences, The University of Tokyo, Tokyo, 113–0033, Japan; 2 Center for Information and Neural Networks, National Institute of Information and Communications Technology, Suita City, Osaka, 565–0871, Japan; Pennsylvania State Hershey College of Medicine, UNITED STATES

## Abstract

Functional multineuron calcium imaging (fMCI) provides a useful experimental platform to simultaneously capture the spatiotemporal patterns of neuronal activity from a large cell population *in situ*. However, fMCI often suffers from low signal-to-noise ratios (S/N). The main factor that causes the low S/N is shot noise that arises from photon detectors. Here, we propose a new denoising procedure, termed the Okada filter, which is designed to reduce shot noise under low S/N conditions, such as fMCI. The core idea of the Okada filter is to replace the fluorescence intensity value of a given frame time with the average of two values at the preceding and following frames unless the focused value is the median among these three values. This process is iterated serially throughout a time-series vector. In fMCI data of hippocampal neurons, the Okada filter rapidly reduces background noise and significantly improves the S/N. The Okada filter is also applicable for reducing shot noise in electrophysiological data and photographs. Finally, the Okada filter can be described using a single continuous differentiable equation based on the logistic function and is thus mathematically tractable.

## Introduction

Functional multineuron calcium imaging (fMCI) is a promising optical technique that simultaneously records suprathreshold activity from a large number of neurons [1–3]. An action potential evokes a transient elevation in intracellular calcium ions in the cell body of neurons. By taking advantage of this phenomenon, fMCI infers the times of action potentials from fluctuations in the fluorescence intensities in neurons that were loaded with calcium-sensitive fluorescent indicators [4]. Typically, an action potential elicits a rapid fluorescence increase that occurs within tens of millisecond, followed by a slow decrease that persists for up to a second [5]. The amplitude of the calcium transients is as small as 2–10% over the baseline intensity when Oregon green 488 BAPTA-1AM (OGB1-AM), the most widely used chemical calcium indicator, is used. Therefore, fMCI is vulnerable to signal-irrelevant noise and often suffers from low signal-to-noise ratios (S/N) [3].

Shot noise is dominant in under low-light intensity conditions during fMCI data acquisition. A major source of shot noise in fMCI is the photodetectors. fMCI mainly uses photomultiplier tubes, charge-coupled devices, or complementary metal-oxide-semiconductors to detect photons. Their signal is inevitably contaminated with internal noise due to the quantal nature of light and the probabilistic nature of photon detection [6]. Thus, the number of photons detected in a given time window could be larger and smaller than the true number, and these stochastic fluctuations cause shot noise.

In fMCI time-series data, shot noise is usually reduced through *post hoc* filtering using a moving window. The filters are classified into two types: linear and nonlinear filters. Most of the linear filters are moving-average low-pass filters with linearly weighted windows, such as rectangular, Gaussian, and Hann windows [7–9]. The basic idea of the linear filters is to replace the value at a focused time with the averaged value over the window. Unfortunately, the linear filters smooth sharply changing values, such as the rising phase of calcium transients, and hinder the precise detection of the onset times of the calcium transients. Moreover, a single outlier due to shot noise affects nearby values within the window. This undesirable effect is relevant in fMCI data that are acquired at sampling rates as low as tens of hertz.

Nonlinear filters may overcome these problems [10]. A widely used nonlinear filter is the median filter [11]. A median filter replaces the value at a focused time with the median value within a moving window. This procedure tends to preserve the structure of a signal; however, unlike linear filters, a median filter selects the replaced values from a pool of values that already existed in the original data, thereby resulting in less efficient denoising.

In this study, we propose a novel nonlinear filter named the Okada filter, which employs the compromise strategies of linear and median filters. The Okada filter replaces the value at a focused time with the average of two values at both ends of a moving window unless the focused value is the median among values in the window. Therefore, the filtered data contain "more centered" values that did not exist in the original values (as in linear filter) and have a greater likelihood of preserving the structure of the putative signal because any values during a consecutive change are unlikely replaced (as occurs when using a median filter).

## Materials and Methods

### Animal ethics

Experiments were performed with the approval of the animal experiment ethics committee at the University of Tokyo (approval number: P24-6) and according to the University of Tokyo guidelines for the care and use of laboratory animals.

### Functional multineuron calcium imaging

Hippocampal organotypic slices were prepared from Wistar/ST rats (SLC), as described elsewhere [12]. Rat pups were anaesthetized via hypothermia and isoflurane and then decapitated. The brains were removed and placed in ice-cold oxygenated Gey’s balanced salt solution supplemented with 25 mM glucose. The brains were sliced horizontally (300 μm thick) using a vibratome (DTK-1500, Dosaka). Then, entorhinal-hippocampal regions were trimmed using a surgical microknife. The slices were placed on Omnipore membrane filters (JHWP02500, Millipore) and incubated in 5% CO_2_ at 35°C. The culture medium, which was composed of 50% minimal essential medium (Invitrogen), 25% Hanks’ balanced salt solution, 25% horse serum (Gibco), and antibiotics, was changed every 3.5 days. Experiments were performed at 11−19 days in vitro. Slices were transferred to a 35-mm dish that was filled with 2 ml of dye solution and incubated for 40 min in a humidified incubator at 37°C in 5% CO_2_ with 0.0005% OGB1-AM (Invitrogen), 0.01% Pluronic F-127 (Invitrogen), and 0.005% Cremophor EL (Sigma-Aldrich) [13]. The slices were then recovered for 30 min in oxygenated artificial cerebrospinal fluid (aCSF) that consisted of (in mM) 127 NaCl, 26 NaHCO_3_, 3.3 KCl, 1.24 KH_2_PO_4_, 1.2 MgSO_4_, 1.2 CaCl_2_, and 10 glucose, which were then bubbled with 95% O_2_ and 5% CO_2_. A slice was mounted in a recording chamber and perfused with aCSF at a rate of 1.5–2.0 ml/min for 15 min. The hippocampal CA3 pyramidal cell layer was imaged at 10 Hz using a Nipkow-disk confocal microscope (CSU-X1, Yokogawa Electric) equipped with a cooled CCD camera (iXonEM+DV897, Andor Technology) and an upright microscope with a water-immersion objective lens (16×, 0.8 numerical aperture, Nikon) [14]. Fluorophores were excited at 488 nm with a laser diode and visualized with a 507-nm long-pass emission filter. The fluorescence change was measured as (*F*_*t*_−*F*_0_)/*F*_0_, where, *F*_*t*_ is the fluorescence intensity at time *t*, and *F*_0_ is the fluorescence intensity averaged from –10 to 10 s relative to *t*.

### Electrical physiological recording

Recordings were performed in a submerged chamber perfused at 6–8 ml/min with oxygenated aCSF at 35°C. Whole-cell patch-clamp recordings were obtained from hippocampal CA3 pyramidal cells that were visually identified under an infrared-differential, interference-contrast microscope. For cell-attached recordings, glass pipettes (3–6 MΩ) were filled with aCSF [15]. For voltage clamp recordings, pipettes were filled with a cesium-based solution that consisted of (in mM) 130 CsMeSO_4_, 10 CsCl, 10 HEPES, 10 creatine phosphate, 4 Mg-ATP and 0.3 Na_2_-GTP [16]. Excitatory postsynaptic currents (EPSCs) were recorded at a clamped voltage of –70 mV. Signals were amplified and digitized at a sampling rate of 2 kHz using a MultiClamp 700B amplifier and a Digidata 1440A digitizer that were controlled by pCLAMP 10.4 software (Molecular Devices).

### Okada filter

The Okada filter is given by the following equation:

$$x_{t}\leftarrow x_{t}+\frac{x_{t-1}+x_{t+1}-2x_{t}}{2\;(1+e^{-\alpha (x_{t}-x_{t-1})(x_{t}-x_{t+1})})}\;,$$

where *x*_*t*–1_, *x*_t_, and *x*_*t*+1_ denote the *ΔF/F* values at frames *t*–1, *t*, and *t*+1, respectively. This equation is based on the logistic function. Therefore, when *α* is sufficiently large, it can perform two processes; (1) if (*x*_*t*_−*x*_*t*–1_)(*x*_*t*_−*x*_*t*+1_) ≤ 0, *x*_*t*_ is substituted with *x*_*t*_ itself, and (2) if (*x*_*t*_−*x*_*t*–1_)(*x*_*t*_−*x*_*t*+1_) > 0, *x*_*t*_ is substituted with (*x*_*t*–1_ + *x*_*t*+1_)/2. This process is repeated subsequently from the second frame to the penultimate frame, whereas the values from the first frame and the last frame are not changed. All routines were written in MATLAB (The MathWorks).

### Median filter

Median filter replaces *x*_*t*_ with the median value within *x*_*t*–1_, *x*_*t*_ and *x*_*t*+1_. This process is repeated subsequently from the second frame to the penultimate frame, whereas the values from the first frame and the last frame are not changed. Unlike the Okada filter, the median filter uses the original *x*_*t*–1_ before substitution as *x*_*t*–1_.

### Binomial filter

Binomial filter replaces *x*_*t*_ with a binomial weighted convolution of *x*_*t*–1_, *x*_*t*_ and *x*_*t*+1_ (here, *x*_*t*_ ← 0.25 *x*_*t*–1_ + 0.5 *x*_*t*_ + 0.25 *x*_*t*+1_). This replacement is repeated subsequently from the second frame to the penultimate frame, whereas the values from the first frame and the last frame are not changed. Unlike the Okada filter, the binomial filter uses the original *x*_*t*–1_ before substitution as *x*_*t*–1_.

### Savitzky-Golay filter

Savitzky-Golay filter replaces *x*_*t*_ with the approximate value estimated by fitting a low order polynomial to a series of {*x*_*t*–1_, *x*_*t*_, *x*_*t*+1_} using the least squared method. This process is repeated subsequently from the second frame to the penultimate frame, whereas the values from the first frame and the last frame are not changed. Unlike the Okada filter, the Savitzky-Golay filter uses the original *x*_*t*–1_ before substitution as *x*_*t*–1_.

### Photograph with shot noise

A portrait of Lenna was downloaded from the Standard Image Data-Base. Shot noise was added with a Poisson distribution as follows:

$$x_{i,j}\;=\;\frac{1}{\text{poissrnd}(x_{i,j})}\;,$$

where *x*_*i*,*j*_ represents the value of the pixel at row *i* and line *j*. This process was applied to all pixels of the photograph.

### Peak signal to noise ratio

The peak signal-to-noise ratio (PSNR) is defined using the mean squared error (MSE) as follows:

$$\text{PSNR}=\;10\;\text{log}\frac{p^{2}}{MSE}\;,$$

where *p* is the maximum luminance value, *i*.*e*., 255 for an 8-bit image or 65,025 for a 16-bit image. When the m×n original image (*I*) was compared to its filtered image (*K*), MSE is given by the following equation:

$$\text{MSE}=\frac{1}{mn}\sum_{i=1}^{m}\sum_{j=1}^{n}{\left(I(i,j)-K(i,j)\right)}^{2}\;.$$

### Frequency response

The power spectrum is computed using the Fourier transformation function with a 10-s hamming window. The ratio of the power of the filtered trace to that of the original trace at a given frequency is defined as a frequency response.

### Statistics

Summarized data are reported as the means ± standard deviations (SDs). For each cell, the logarithm of the mean amplitude of calcium transients divided by the SD of the fluorescence intensities during baseline periods, except ± 0.5 s relative to the peak of calcium transient, was calculated as the S/N. Wilcoxon signed rank test and paired *t*-test were performed to assess the significance of the differences. *P*<0.05 was considered statistically significant.

## Results

### Okada filter

**Fig 1:**
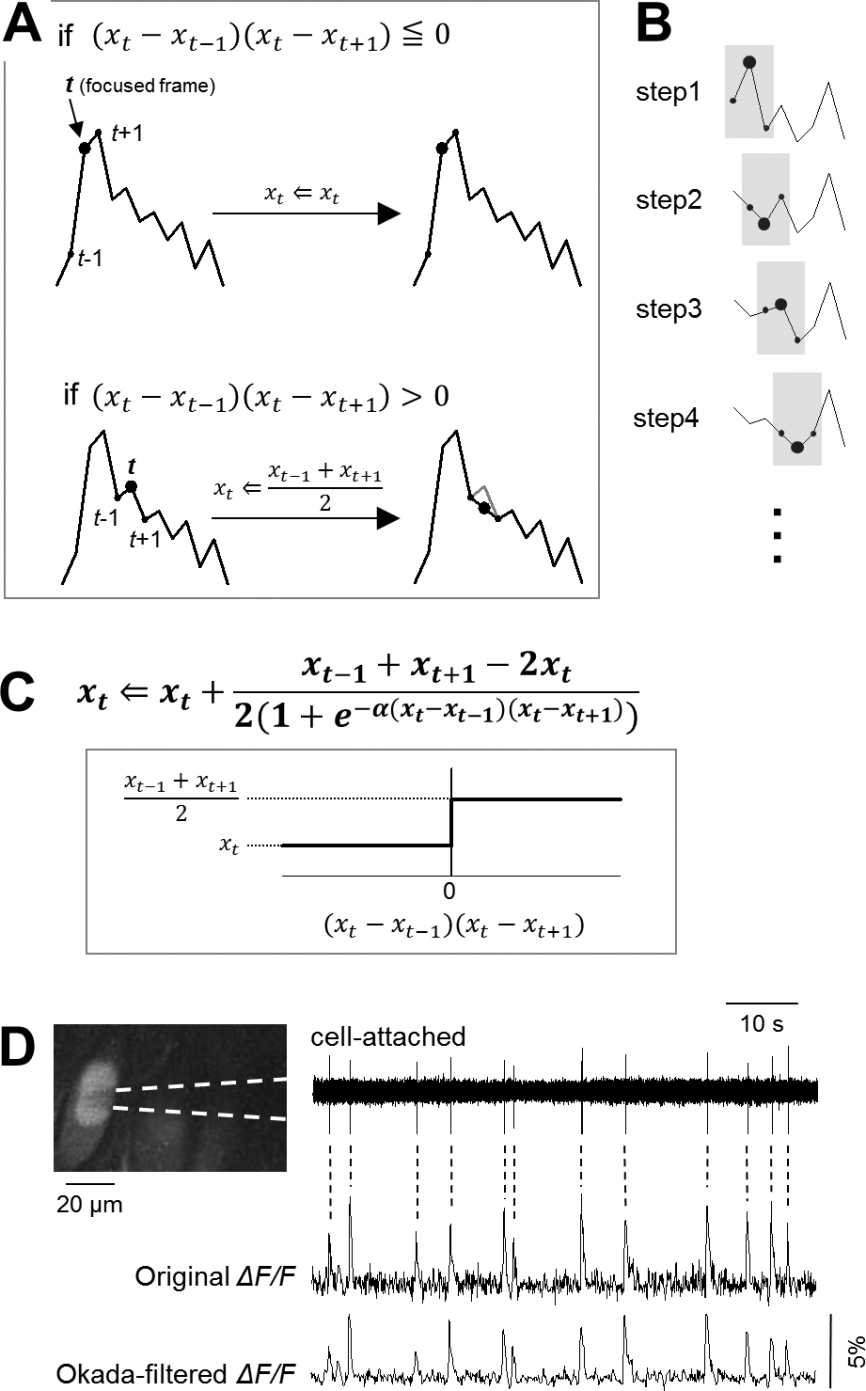
Introduction of the Okada filter. **A.** Schematic illustration for data processing of the Okada filter. At the focused time *t*, *x*_*t*_ is compared with the preceding and following values *x*_*t*–1_ and *x*_*t*+1_, respectively. If *x*_*t*_ is the median among *x*_*t*–1_, *x*_*t*_, and *x*_*t*+1_, then *x*_*t*_ is not modified (top). If *x*_*t*_ is not the median, then *x*_*t*_ is substituted with the mean of *x*_*t*–1_ and *x*_*t*+1_, *i*.*e*., (*x*_*t*–1_ + *x*_*t*+1_)/2 (bottom). **B.** The substitution procedure in A is serially conducted from *t* = 2 to the number of data points − 1. In each step, the value updated in the preceding step is used as *x*_*t*–1_. **C.** The Okada filter is expressed in a single equation based on the logistic function. The coefficient α determines the steepness in the transition at (*x*_*t*_ − *x*_*t*–1_) (*x*_*t*_ − *x*_*t*+1_) = 0. If α is more than one order of magnitude higher than the maximal value in {*x*_*t*_}, the transition can be regarded as a digital-like jump. **D.** Simultaneous cell-attached unit recording (top) and fMCI from a CA3 pyramidal cell (middle). The fMCI trace was filtered using the Okada filter with α = 100 (bottom). Dashed lines represent the times of action potentials detected in cell-attached trace.

**Fig 2:**
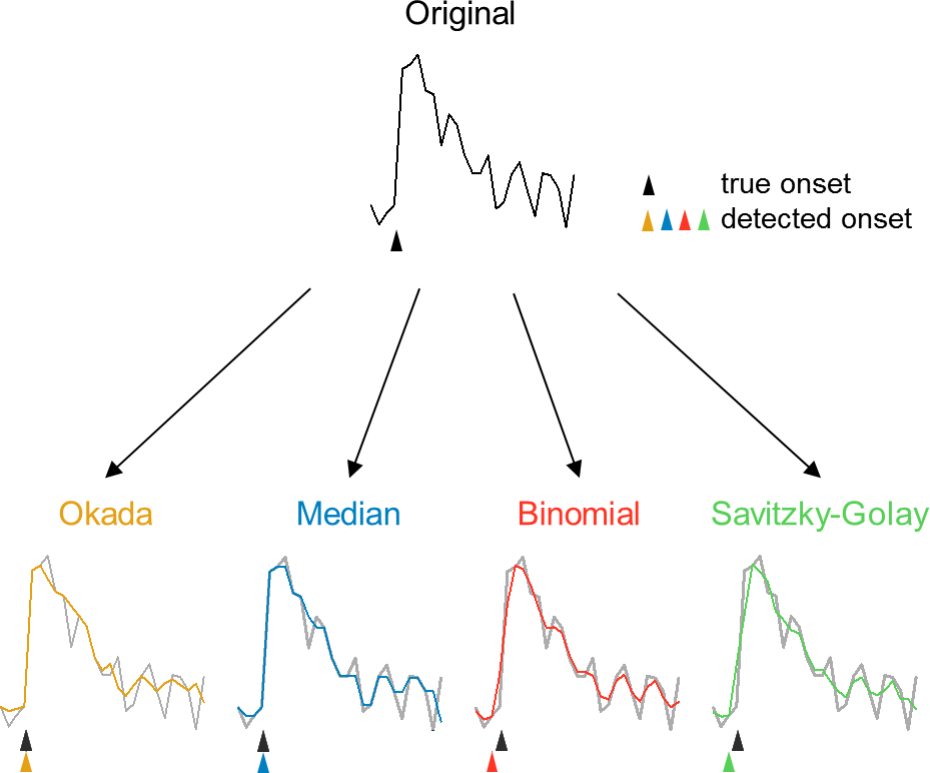
The Okada filter does not burr the signal onsets. The original fMCI trace is shown in the top (black), and the black arrowhead indicates the true onset of the calcium transient. In the bottom, each trace after the Okada (orange), median (blue), binomial (red), or Savitzky-Golay (green) filter were superimposed onto the original trace (gray). The colored arrowheads indicate the "detected" onsets of the calcium transient in the filtered traces. Note that both the binomial and Savitzky-Golay filters caused an erroneous shift in the onset time.

The Okada filter is designed to remove outliers in a given time-series vector {*x*_*t*_} (*t* ranges from 1 to T). At each step at time *t*, the Okada filter compares *x*_*t*_ to the immediately preceding and following values, *x*_*t*–1_ and *x*_*t*+1_, respectively. If *x*_*t*_ is the median among *x*_*t*–1_, *x*_*t*_, and *x*_*t*+1_, that is, if (*x*_*t*_*−x*_*t*–1_)(*x*_*t*_−*x*_*t*+1_) ≤0, then *x*_*t*_ is not changed. If *x*_*t*_ is not the median, that is, if (*x*_*t*_−*x*_*t*–1_)(*x*_*t*_*−x*_*t*+1_) >0, then *x*_*t*_ is substituted with the average of *x*_*t*–1_ and *x*_*t*+1_, *i*.*e*., (*x*_*t*–1_ + *x*_*t*+1_)/2 (Fig 1A). This procedure is conducted sequentially from *t* = 2 to T– 1 (Fig 1B). In each step, the value that was updated in the preceding step is used as *x*_*t*–1_. Notably, the substitution process can be expressed by a single equation using a logistic function (Fig 1C). When the coefficient α is sufficiently large, the equation takes either *x*_*t*_ or (*x*_*t*–1_ + *x*_*t*+1_)/2, depending on the sign of (*x*_*t*_*−x*_*t*–1_)(*x*_*t*_*−x*_*t*+1_). Specifically, if (*x*_*t*_*−x*_*t*–1_)(*x*_*t*_*−x*_*t*+1_) is negative, the equation substitutes *x*_*t*_ with the same value *x*_*t*_, otherwise the equation substitutes *x*_*t*_ with (*x*_*t*–1_ + *x*_*t*+1_)/2. This conditional substitution is aimed to filter out a sudden outlier, such as shot noise, in the time-series vector {*x*_*t*_} without affecting a continuous increase or decrease in {*x*_*t*_} because the latter might reflect signal, such as the onset of a calcium transient. This conditional process is important to preserve the waveform of signal; note that wildly used filters, such binomial and Savitzky-Golay filters, inevitably smooth the waveforms of sharp signal (e.g., calcium transients in fMCI data) and may lead to erroneous detections of the exact timings of signal onsets (Fig 2). Nonlinear filters, such as the Okada filter and median filter preserve the true signal onsets (Fig 2).

### Filtering fMCI data

**Fig 3:**
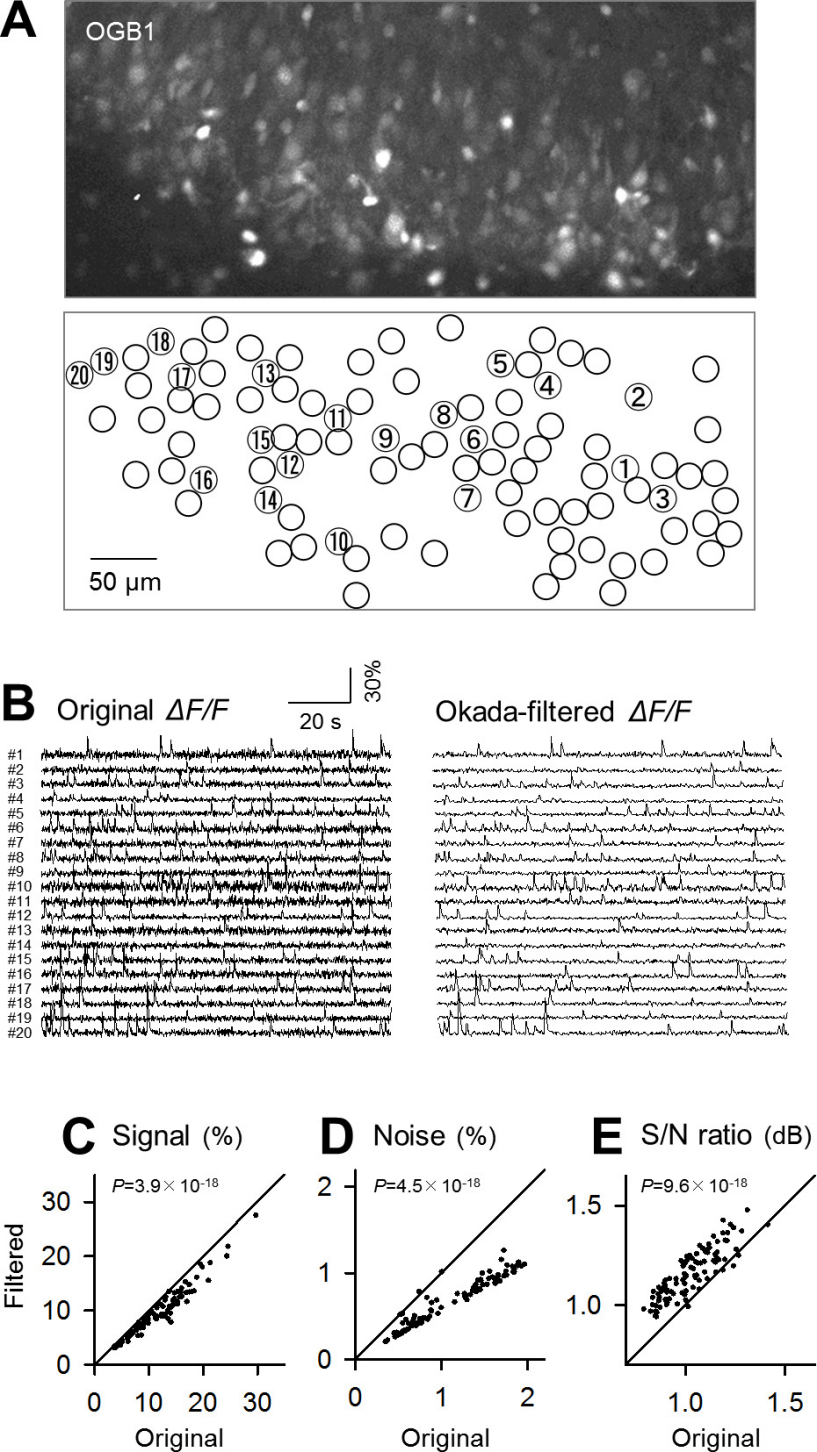
Application of the Okada filter to fMCI data. **A.** In a confocal image of the CA3 region of cultured hippocampal slices loaded with OGB1-AM (top), the cell bodies of neurons were identified (bottom). **B.** Left 20 traces are raw fluctuations in the OGB1 fluorescence intensities in the cell bodies numbered in **A** and were Okada-filtered (right). **C.** Comparison of the mean signal, the mean amplitude of calcium transients that occurred in individual cells, before (abscissa) and after Okada filtering (ordinary). Each dot indicates a single cell. **D.** The same as **C**, but for the background noise level, the SDs in fluorescence intensities during the baseline period in the absence of calcium transients. **E.** The same as **C**, but for the S/N ratio, the mean signal divided by the background noise level. *P* was determined using Wilcoxon signed rank test for 100 cells.

We applied the Okada filter to fMCI data. The fluorescence intensities were measured from the cell bodies from CA3 neurons in organotypic hippocampal slice cultures loaded with OGB1-AM. Because the amplitudes of calcium transients were maximally ~10%, the constant α was set to 100. The Okada filter reduced noise from the original calcium trace, embossing the waveforms of spike-evoked calcium transients (Fig 1D). Other examples are shown in Fig 3A and 3B, in which the calcium traces of 20 representative neurons were filtered using the Okada filter.

We sought to quantify the denoising power of the Okada filter. We randomly sampled 100 cells from our laboratory data storage and applied the Okada filter. Then, the levels of signal and noise and their ratios (S/N) were compared before and after the filtering (Fig 3C–3E). The mean amplitudes of calcium transients (signal) were significantly reduced in the Okada-filtered traces compared to the original traces (Fig 3C, *P* = 3.9×10^−18^, *Z* = 8.68, Wilcoxon signed rank test, *n* = 100 cells). This undesirable signal reduction resulted from substitutions of the peak values *x*_*t*_ of calcium transients with (*x*_*t*–1_ + *x*_*t*+1_)/2. Noise was quantified as the SD of the fluctuations in fluorescence during the baseline periods without calcium transients. Noise was significantly reduced in Okada-filtered trace (Fig 3D, *P* = 4.5×10^−18^, *Z* = 8.67). We calculated the S/N by dividing the mean amplitudes of calcium transients (signal) by the SD of the baseline fluorescence fluctuations (noise). The S/N of the Okada-filtered traces was significantly higher than that of the original traces (Fig 3E, *P* = 9.6×10^−18^, *Z* = 8.58), indicating that the Okada filter reduces noise to a greater degree than the signal.

### Comparison of denoising power

**Fig 4:**
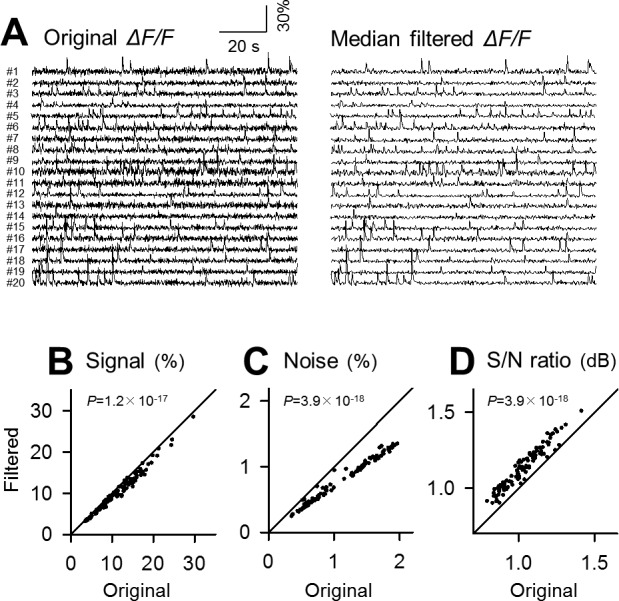
Application of the median filter to fMCI data. **A.** Left 20 traces are raw fluctuations in the OGB1 fluorescence intensities in the cell bodies numbered in Fig 3A and were median-filtered (right). **B-D.** Comparison of the signal (B), the background noise level (B) and its ratio (D) before and after median filtering. Each dot indicates a single cell. *P* was determined using Wilcoxon signed rank test for the same 100 cells as those used in Fig 3C–3E.

**Fig 5:**
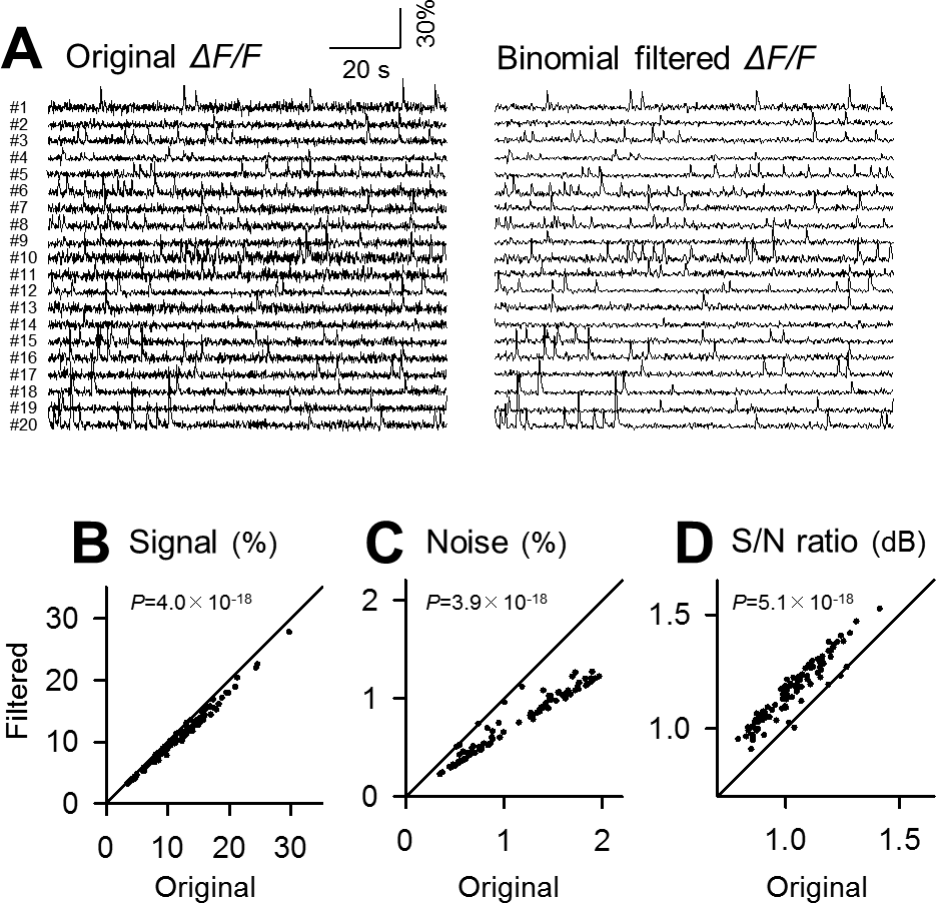
Application of the binomial filter to fMCI data. **A**. Left 20 traces are raw fluctuations in the OGB1 fluorescence intensities in the cell bodies numbered in Fig 3A and were binomial-filtered (right). **B**-**D**. Comparison of the signal (B), the background noise level (C) and its ratio (D) before and after binomial filtering. Each dot indicates a single cell. *P* was determined using Wilcoxon signed rank test for the same 100 cells as those used in Fig 3C–3E.

**Fig 6:**
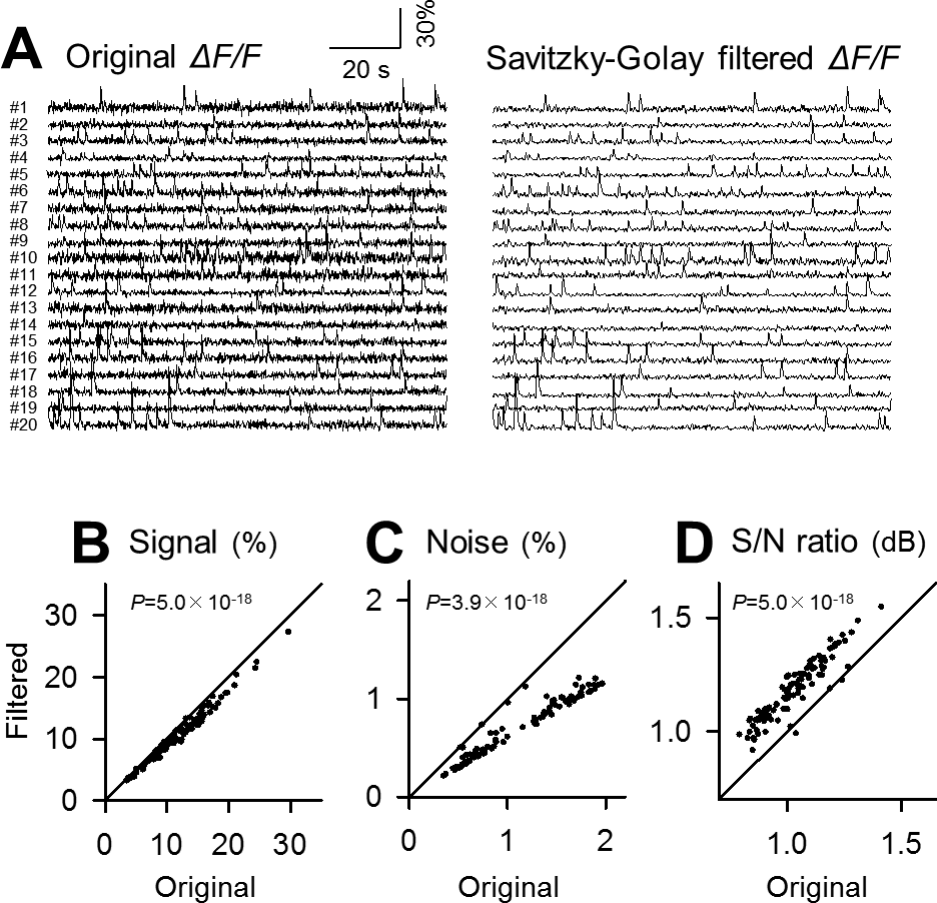
Application of the Savitzky-Golay filter to fMCI data. **A**. Left 20 traces are raw fluctuations in the OGB1 fluorescence intensities in the cell bodies numbered in Fig 3A and were Savitzky-Golay-filtered (right). **B**-**D**. Comparison of the signal (B), the background noise level (C) and its ratio (D) before and after Savitzky-Golay filtering. Each dot indicates a single cell. *P* was determined using Wilcoxon signed rank test for the same 100 cells as those used in Fig 3C–3E.

To estimate the merit of the Okada filter, we repeated the same analysis for the median, binomial and Savitzky-Golay filters (Figs 4A, 5A and 6A; *n* = 100 cells). To facilitate comparisons, the identical datasets were filtered. All these filters also reduced the signal and noise (Fig 4B, *P* = 1.2×10^−17^, *Z* = 8.55; Fig 4C, *P* = 3.9×10^−18^, *Z* = 8.68; Fig 5B, *P* = 4.0×10^−18^, *Z* = 8.68; Fig 5C, *P* = 3.9×10^−18^, *Z* = 8.68; Fig 6B, *P* = 5.0×10^−18^, *Z* = 8.65; Fig 6C, *P* = 3.9×10^−18^, *Z* = 8.68) and increased the S/N (Fig 4D, *P* = 3.9×10^−18^, *Z* = 8.68; Fig 5D, *P* = 5.1×10^−18^, *Z* = 8.65; Fig 6D, *P* = 5.1×10^−18^, *Z* = 8.65).

**Fig 7:**
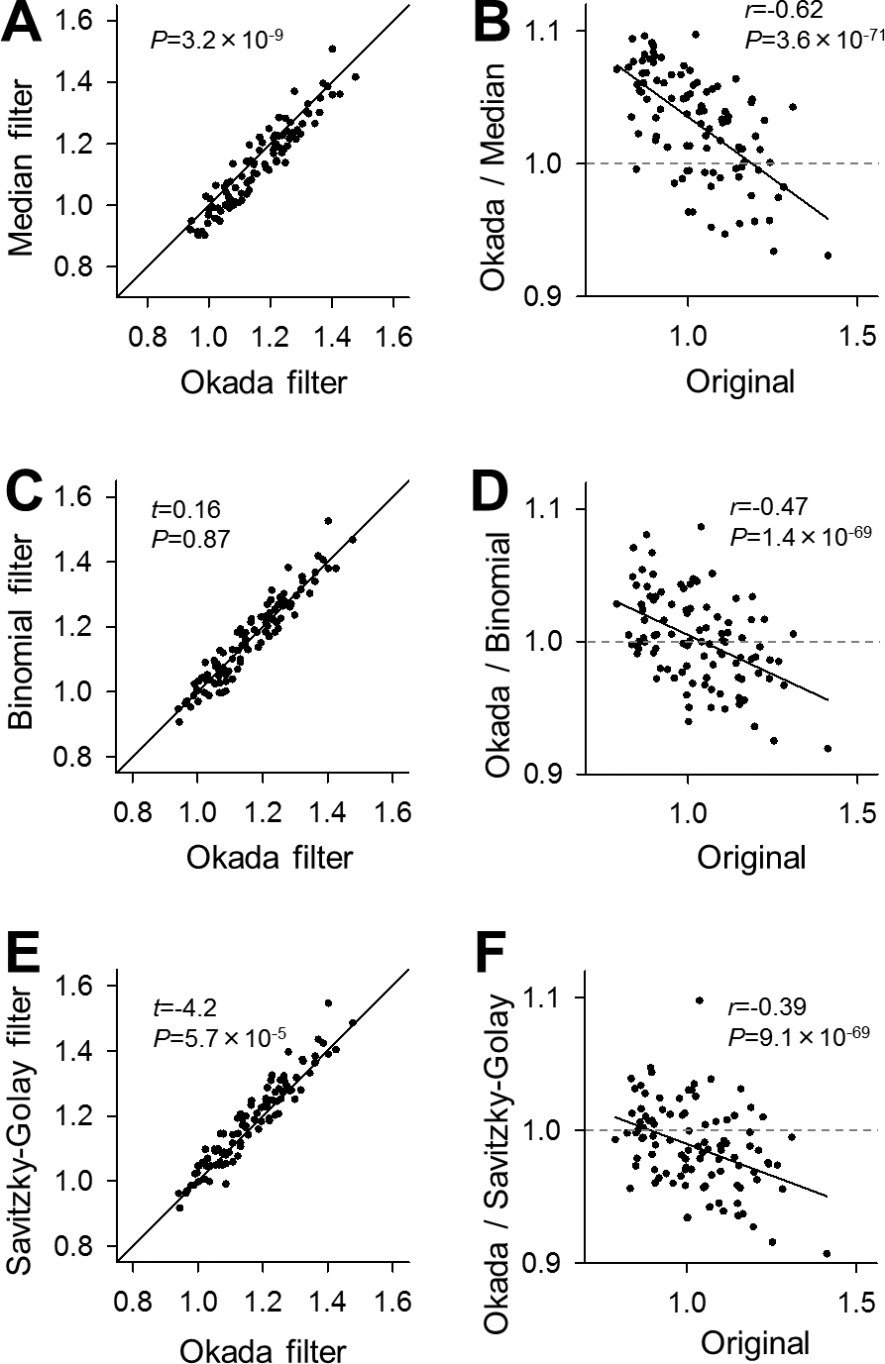
Comparison of S/N improved by the Okada and the median, binomial, and Savitzky-Golay filters. **A.** The S/N is compared between Okada-filtered (abscissa) and median-filtered traces (ordinary). Each dot represents a single cell. Wilcoxon signed rank test. *n* = 100 cells. **B.** The ratios of the S/N in the Okada-filtered traces to those in median-filtered traces are plotted against the S/N in the original raw traces. Pearson’s correlation coefficient *r* was negative, and the regression line crossed *y* = 1 at the original S/N of 1.19. **C**. Same as **A**, but compared to the binomial-filtered traces. **D**. Same as **B**, but for binomial-filtered traces. Pearson’s correlation coefficient *r* was negative, and the regression line crossed *y* = 1 at the original S/N of 1.04. **E**. Same as **A**, but compared to Savitzky-Golay-filtered traces. **F**. Same as **B**, but for Savitzky-Golay-filtered traces. Pearson’s correlation coefficient *r* was negative, and the regression line crossed *y* = 1 at the original S/N of 0.90.

We compared the S/N of the Okada-filtered and median filtered traces (Fig 7A). The S/N varied among datasets; however, as a whole, the Okada filter produced significantly higher S/N than did the median filter (Fig 7A, *P* = 3.2×10^−9^, *Z* = 5.92). This difference appeared to be due to the greater ability of the Okada filter to remove baseline noise. Therefore, to examine whether the noise level affected the performance of these filters, we plotted the ratios of the S/N of Okada-filtered traces to those of median-filtered traces against the S/N of the original traces. These parameters exhibited a negative correlation (Fig 7B, Pearson’s *r* = −0.62, *P* = 3.6×10^−71^, *t*_98_ = 50), and the regression line crossed *y* = 1 at the S/N of 1.19 dB. Thus, the Okada filter removed noise more efficiently from the lower S/N data. We also compared the S/N of the Okada-filtered traces with the binomial-filtered and Savitzky-Golay-filtered traces in the same manner. The Okada filter improved the S/N to a degree that was comparable to the binomial filter (Fig 7C, *t* = 0.16, *P* = 0.87, paired *t*-test) but that was lower than the Savitzky-Golay filter (Fig 7E, *t* = 4.2, *P* = 5.7×10^−5^). The high performances of binomial and Savitzky-Golay filters were presumably because these filters preserve the signal amplitude more than the Okada filter. However, like the Fig 7B plot, the Fig 7D and 7F plots also exhibited a negative correlation (Fig 7D, Pearson’s *r* = −0.47, *P* = 1.4×10^−69^, *t*_98_ = 48; Fig 7F, Pearson’s *r* = −0.39, *P* = 9.1×10^−69^, *t*_98_ = 47). These results confirm that the Okada filter worked relatively efficiently in the lower S/N data.

**Fig 8:**
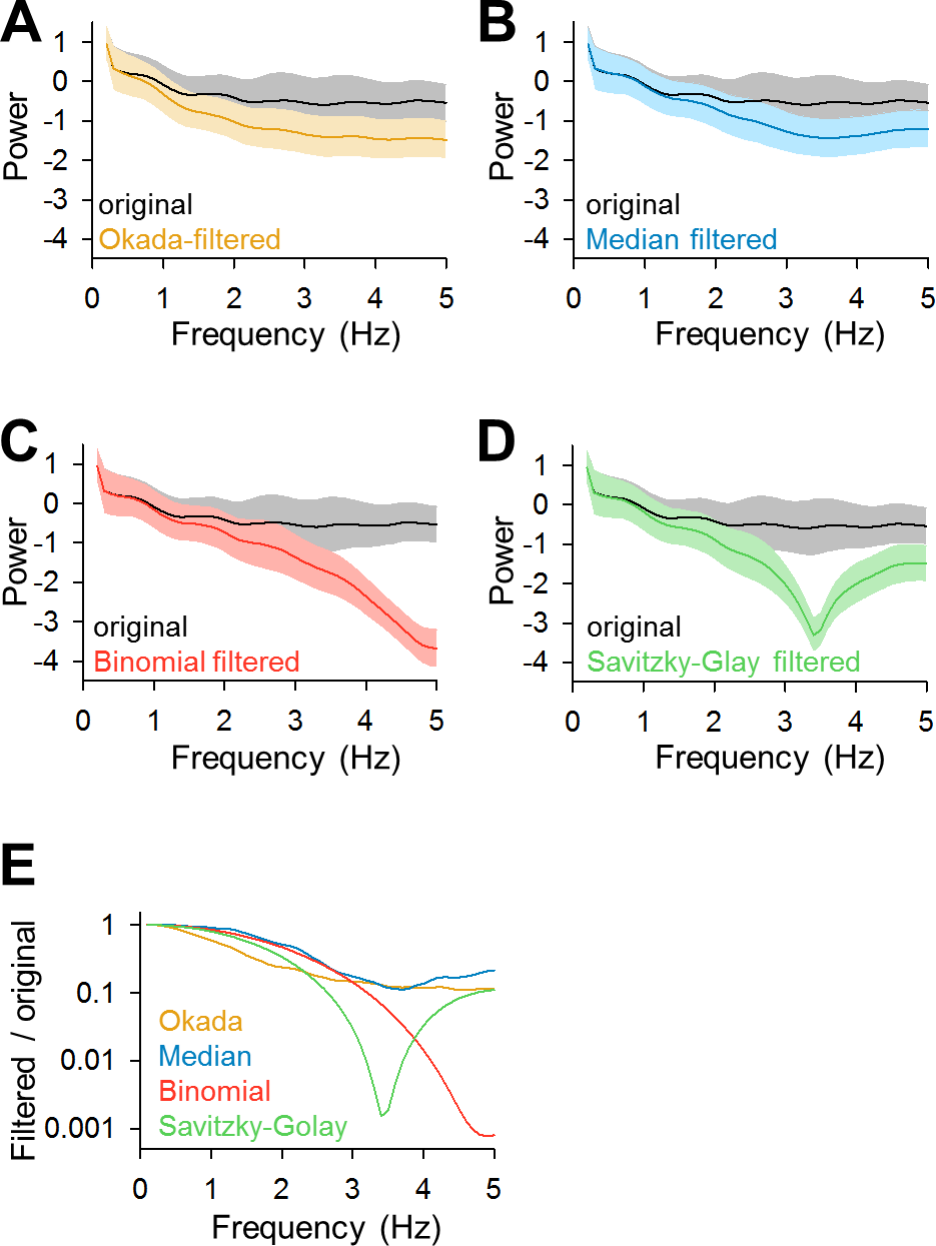
Comparisons of the frequency responses of the Okada, median, binomial, and Savitzky-Golay filters. **A**. The mean ± SD power spectra of the 100 cells data same as used in Fig 3 are shown in black, where those of Okada-filtered traces are shown in color. **B**-**D**. Same as **A**, but for the median (**B**, blue), binomial (**C**, red) and Savitzky-Golay (**D**, green) filtered traces. **E**. The mean frequency response of each filter is shown relative to the mean powers of the original traces.

We computed the power spectra of the original traces and the traces that were filtered by the Okada, median, binomial and Savitzky-Golay filters (Fig 8A–8D, *n* = 100). Then we plotted the frequency responses, the ratios of the powers of the filtered traces to that of the original traces (Fig 8E). The Okada and median filters broadly reduced the power with a low-pass trend, whereas the binominal and Savitzky-Golay filters reduced the power in a more frequency-specific manner.

### Comparison of computational speed

**Fig 9:**
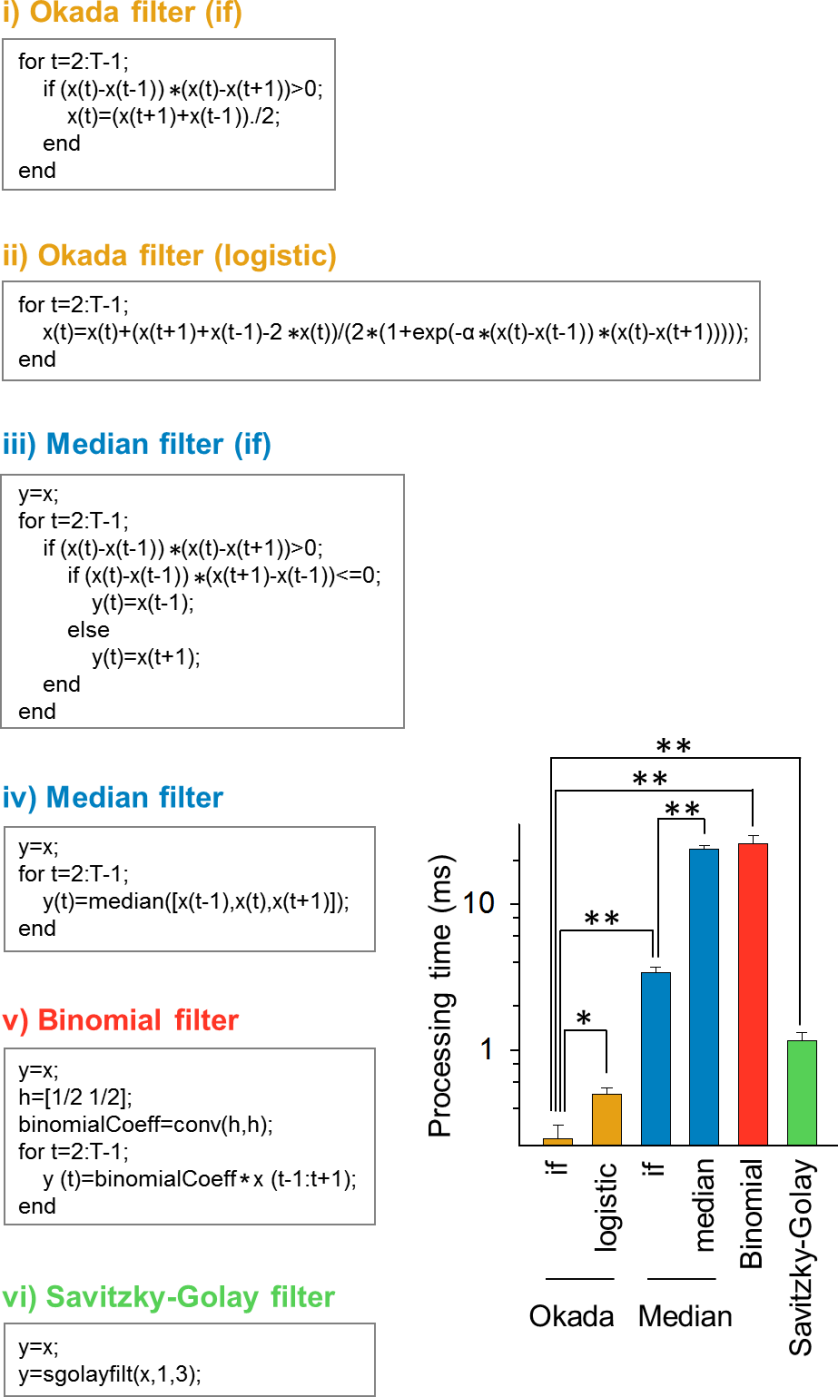
Comparison of computational speed of the Okada and median, binomial, and Savitzky-Golay filters. The statement of each left box is the MATLAB command lines executed in the corresponding filter. Note that the statements i) and ii) and the statements iii) and iv) are theoretically identical but are differently described. The bar graph indicates the mean ± SD times spend in computing in these MATLAB codes. *P = 2.1×10^−17^, **P = 3.9×10^−18^; Wilcoxon signed rank test. *n* = 100 cells.

We next measured the computing speed of the Okada filter. We used a commercially available laptop computer equipped with Intel® Core™ i5-3337U Processor (64 bit, 1.80 GHz) and 8.0 GB RAM. Using the MATLAB code based the 'if/else' statement in Fig 9i), we filtered 100 calcium traces used above. These data were taken at 10 frames per second for 300 s and thus contained 3,000 data points. The computation time spent to filter a single trace was, on average, 247 ± 58 μs (mean ± SD of 100 cells). The Okada filter can be expressed using the logistic function (Fig 9ii). That the commands executed in Fig 9i) and 9ii) are theoretically identical, but the computation time of Fig 9ii) was 499 ± 51 μs and was twice longer than that of Fig 9i) (*P* = 2.1×10^−17^, *Z* = 8.49, Wilcoxon signed rank test, *n* = 100). This difference is probably because the exponential function demanded a higher computational cost. We also measured the computational speed of the median filter. Fig 9iii) uses the 'if' statement to examine whether *x*_*t*_ is the median. Its computational time was 3.76 ± 0.85 ms and was approximately 14 times longer than that of Fig 9i) (*P* = 3.9×10^−18^, *Z* = 8.68). If we used a MATLAB function 'median', the computational time increased further to 23.7 ± 1.6 ms. Moreover, we examined the computational speed of the binomial and Savitzky-Golay filters using the MATLAB codes described in Fig 9v) and vi). Their computational times were 26.0 ± 3.4 ms and 1.17 ± 0.15 ms, respectively, and were both significantly longer than the Okada filter (both *P* = 3.9×10^−18^, *Z* = 8.68). Thus, the Okada filter is superior to the other filters in terms of computational speed. The rapid computation of the Okada filter is mainly due to the fact that it can skips the substituting process when the focused value *x*_*t*_ is the median; note that the linear filter has to update all values in the vector.

### Effect of the window length of Okada filter on denoising performance

**Fig 10:**
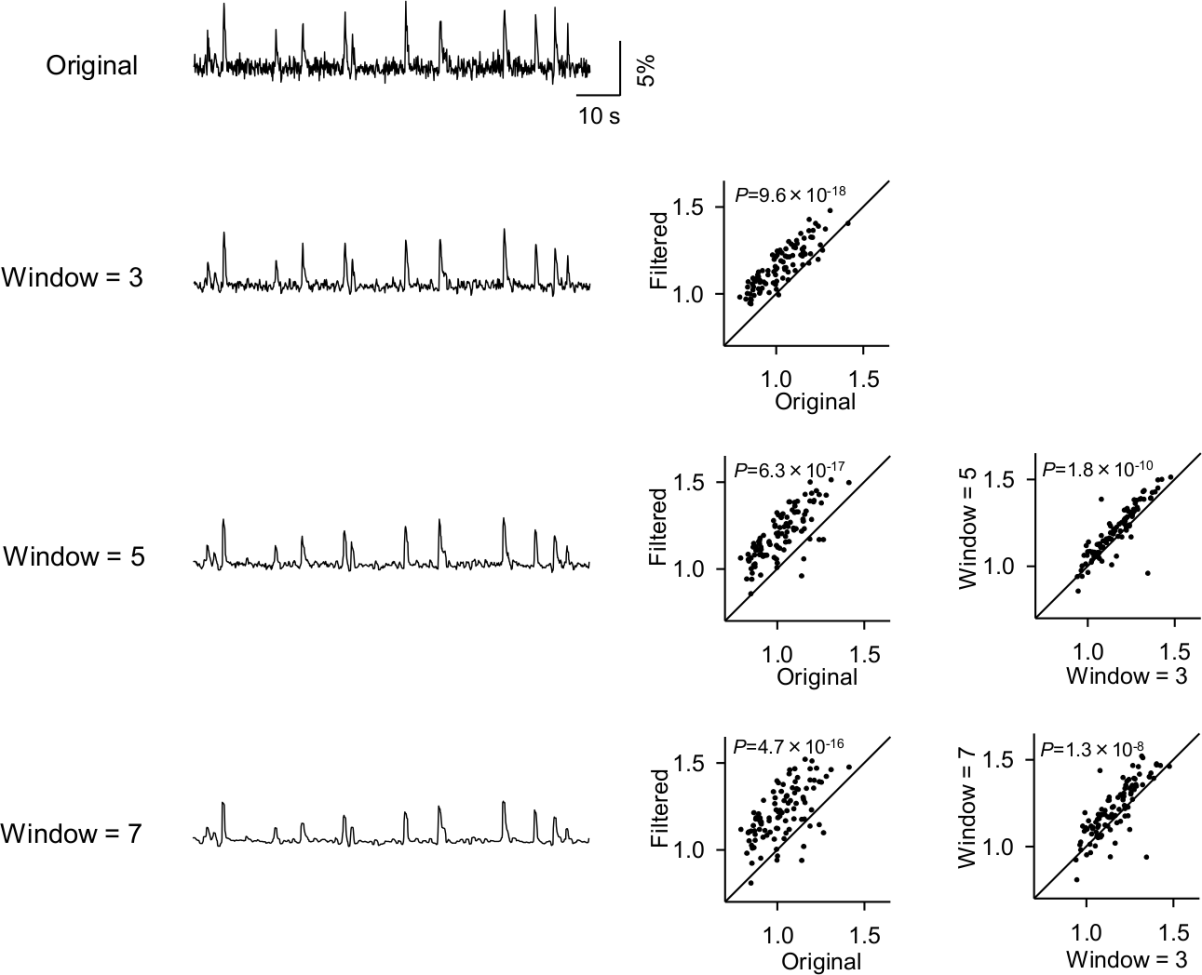
Application of the Okada filter with different window lengths to fMCI data. The original fMCI trace (top) was filtered by the Okada filters with window lengths of three, five and seven frames (left). The same fMCI data as Fig 3 were filtered. The S/Ns in the filtered traces were compared to the original S/Ns (middle), and the effect of the Okada filter with the windows of five and seven frames was compared to that with three frames (right).

In most of the denoising filters, the window length is modifiable. Therefore, we compared the performance of the Okada filters with different window lengths. To preserve the amplitude of signal, we changed the substitutional procedure of the Okada filter. Specifically, the focused value *x*_*t*_ is not changed if it is the median among the values within the window, whereas if *x*_*t*_ is not the median, *x*_*t*_ is substituted with the average of three points around the median, *i*.*e*., the median and two immediately higher and lower values (two nearest neighbor values) of the median within the window. We applied the Okada filters with different window lengths, three, five and seven frames, to fMCI data. In all cases, the filters reduced the noise of traces (Fig 10, left) and improved the S/N (Fig 10, middle, window = 3, *P* = 9.6×10^−18^, *Z* = 8.58, window = 5, *P* = 6.3×10^−17^, *Z* = 8.36, window = 7, *P* = 4.7×10^−16^, *Z* = 8.12, Wilcoxon signed rank test, *n* = 100). Compared to the window of three frames (the original Okada filter), the filter with longer window improved the S/N more effectively (Fig 10, right, window = 5, *P* = 1.8×10^−10^, *Z* = 6.38, window = 7, *P* = 1.3×10^−8^, *Z* = 5.68). Thus, the Okada filter with longer window improved the S/N of fMCI data more efficiently.

### Modified Okada filters

**Fig 11:**
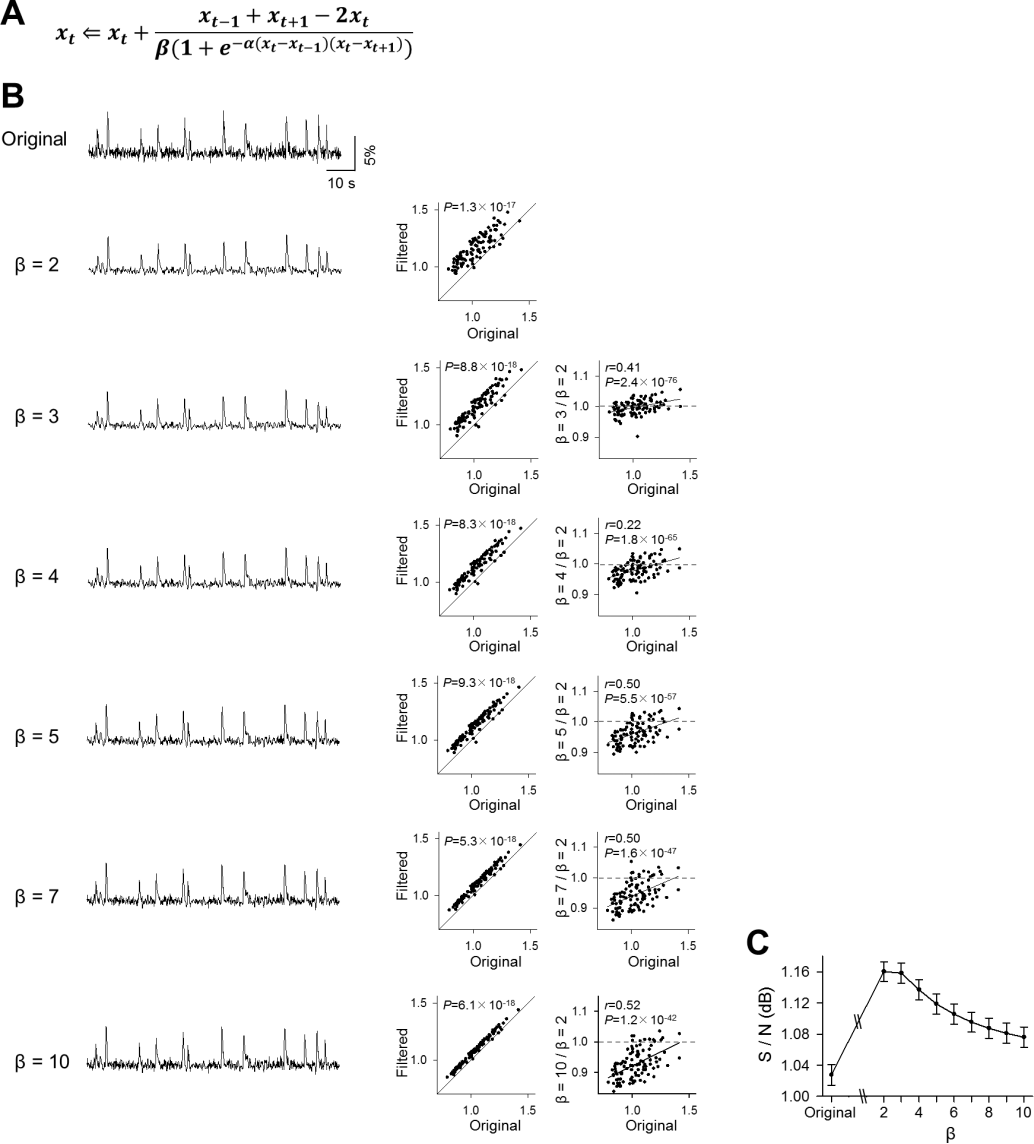
Application of the modified Okada filters to fMCI data. **A**. The modified Okada filter is expressed in the equation similar to that shown in Fig 1C, but the coefficient β in the denominator can take a value different from 2. **B**. The original fMCI trace (top) was filtered by the Okada filter (β = 2) and the modified Okada filters (β = 3–10). The same fMCI data as Fig 3 were filtered. The S/Ns in the filtered traces were compared to the original S/Ns (middle), and the effect of the modified Okada filter was compared to the Okada filter (right). **C**. The mean ± SEM S/Ns of the 100 filtered traces are plotted as a function of β.

The mean filter can be modified to the binomial and Gaussian filters using appropriate weighting factors. Likewise, the Okada filter can also be modified by changing the coefficient β in the equation in Fig 11A; higher β conditions preserve *x*_*t*_ more heavily in the replaced value. That is, the modified Okada filter is expected to work more similarly to a binomial filter when the value replacement occurs. We thus tested the performance of the modified Okada filter with different values of β > 2 (Fig 11B, left). Under all β conditions tested, the modified filter improved the S/N of fMCI data (Fig 11B, middle, β = 2, *P* = 1.3×10^−17^, *Z* = 8.54; β = 3, *P* = 8.8×10^−18^, *Z* = 8.59; β = 4, *P* = 8.3×10^−18^, *Z* = 8.60; β = 5, *P* = 9.3×10^−18^, *Z* = 8.58; β = 6, *P* = 6.1×10^−18^, *Z* = 8.63; β = 7, *P* = 5.3×10^−18^, *Z* = 8.63; β = 8, *P* = 6.1×10^−18^, *Z* = 8.63; β = 9, *P* = 6.1×10^−18^, *Z* = 8.63; β = 10, *P* = 6.1×10^−18^, *Z* = 8.63, Wilcoxon signed rank test, *n* = 100). Compared to the original Okada filter (β = 2), the effect of the modified Okada filters with higher β values were less sufficient to the low S/N data (Fig 11B, right, β = 3, Pearson’s *r* = 0.41, *P* = 2.4×10^−76^, *t*_98_ = 56; β = 4, *r* = 0.22, *P* = 1.8×10^−65^, *t*_98_ = 43; β = 5, *r* = 0.50, *P* = 5.5×10^−57^, *t*_98_ = 35; β = 6, *r* = 0.51, *P* = 2.0×10^−52^, *t*_98_ = 31; β = 7, *r* = 0.50, *P* = 1.6×10^−47^, *t*_98_ = 27; β = 8, *r* = 0.52, *P* = 4.0×10^−46^, *t*_98_ = 26; β = 9, *r* = 0.52, *P* = 3.6×10^−44^, *t*_98_ = 25; β = 10, *r* = 0.52, *P* = 1.2×10^−42^, *t*_98_ = 24). As a result, the S/N of the filtered traces were the highest at β = 2 (the Okada filter) and were decreased with larger β (Fig 11C).

### Further applications of the Okada filter

Although we originally developed the Okada filter to denoise vector sequences with low S/N and with low temporal resolution, such as fMCI data, we finally tried to apply it to other forms of data.

**Fig 12:**
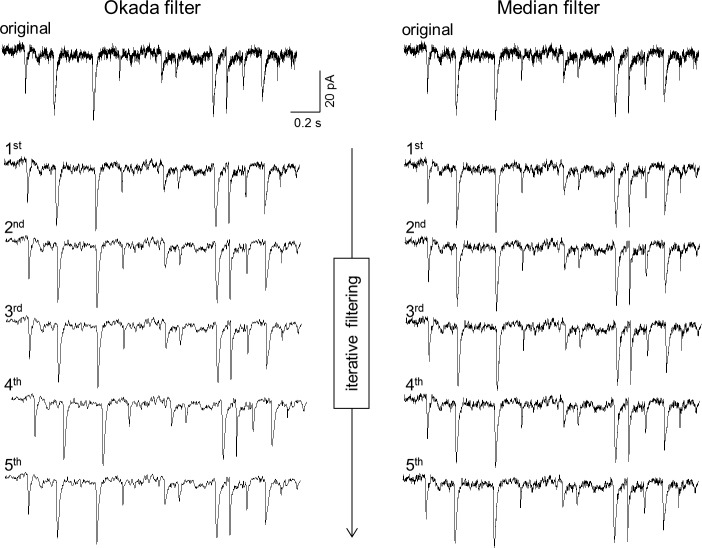
Application of the Okada and median filters to electrophysiological data. A raw EPSC trace acquired using whole-cell voltage-clamped recording at 2 kHz (top) was repeatedly filtered up to five times using the Okada (left) and median filters (right). Note that the background noise is reduced even more when the Okada filtering is repeated more.

We first filtered electrophysiological data acquired at a high temporal resolution (Fig 12). Excitatory postsynaptic currents were recorded at 2 kHz from CA3 pyramidal cells in a hippocampal slice culture. The Okada filter and the median filter both reduced the baseline noise; however, there was still noise in the filtered traces. We thus repetitively applied the filters and found a critical difference in the denoising performance between the Okada filter and the median filter, particularly in later stages of the iterations. Noise was gradually reduced when the Okada filter was repeated, but the median filter did not show this effect. The Okada filter fundamentally substitutes *x*_*t*_ with a new value that is different from *x*_*t*–1_, *x*_*t*_, or *x*_*t*+1_. Conversely, the median filter necessarily selects a substituted value from either *x*_*t*–1_, *x*_*t*_, or *x*_*t*+1_, and thus, the effect of iterative filtering is limited.

We also investigated the effect of the Okada filter on photo images with shot noise. The Okada filter compares values of three neighboring points in a one-dimensional vector {*x*_*t*_}, and this idea cannot be applied to two-dimensional (2D) matrix {*x*_*i*,*j*_}. Therefore, we made a small modification. The 2D-modified Okada filter substitutes *x*_*i*,*j*_ with the average of the vertically and horizontally scanned Okada-filtered values. This process is expressed by the following equation:

$$x_{i,j}\leftarrow x_{i,j}+\left[\frac{x_{i-1,j}+x_{i+1,j}-2x_{i,j}}{2\left\{1+e^{-\alpha \left(x_{i,j}-x_{i-1,j}\right)\left(x_{i,j}-x_{i+1,j}\right)}\right\}}+\frac{x_{i,j-1}+x_{i,j+1}-2x_{i,j}}{2\left\{1+e^{-\alpha \left(x_{i,j}-x_{i,j-1}\right)\left(x_{i,j}-x_{i,j+1}\right)}\right\}}\right]/2\;.$$

**Fig 13:**
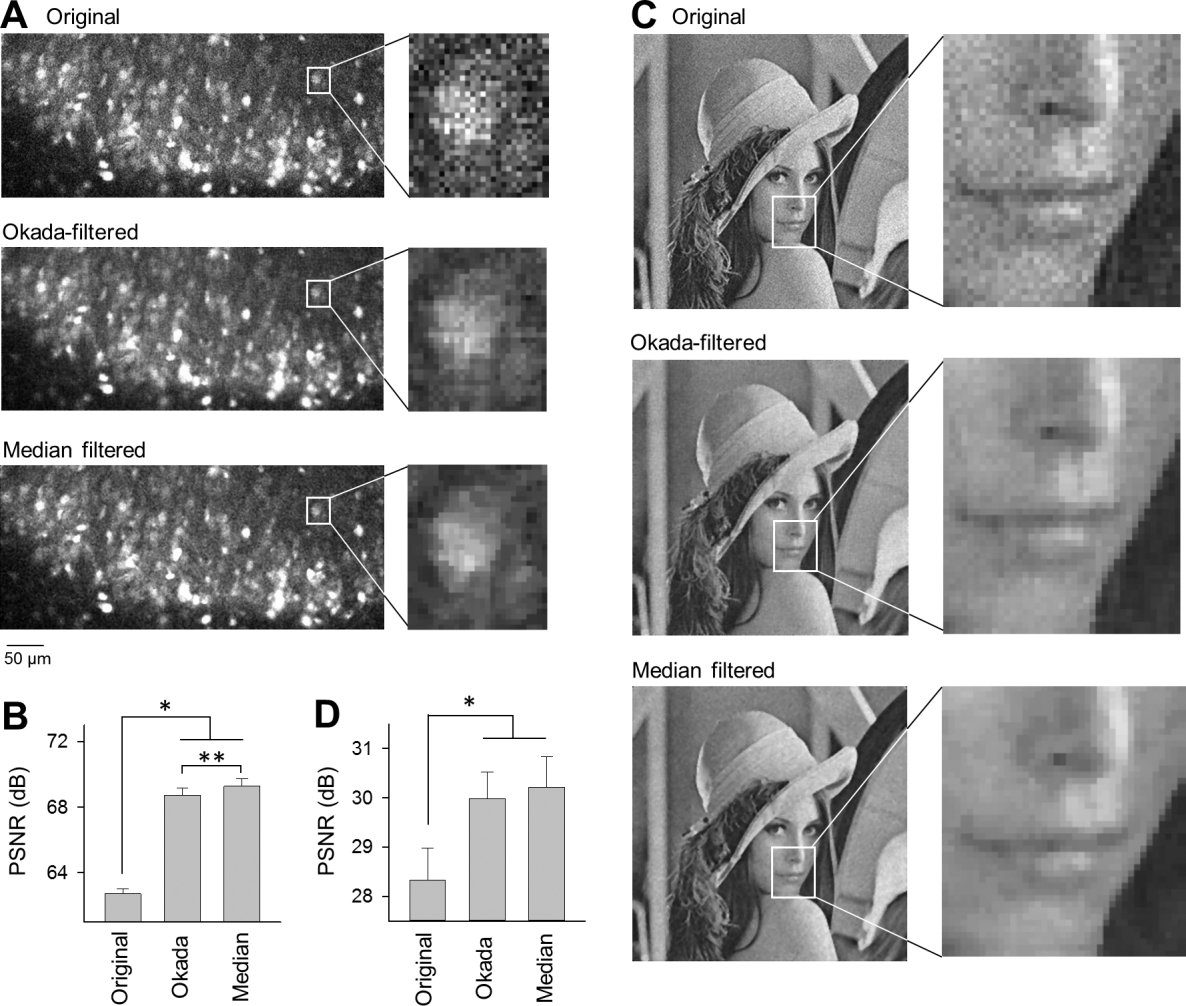
Application of the Okada and median filters to photographs. **A.** A single frame of fMCI (top) was Okada-filtered (middle) and median-filtered (bottom). **B.** The PSNR was compared between the original image and the Okada-filtered or median-filtered images shown in **A**. *P = 1.8×10^−5^, *Z* = 4.29, Wilcoxon signed rank test (*F*_23,23_ = 2.13, 2.20, *F*-test of equality of variances); **P = 4.5×10^−12^, *t*_23_ = 13.0, paired *t*-test (*F*_23,23_ = 1.03, *F*-test of equality of variances), mean ± SEM of 24 subregions. **C**. The same as **A**, but for a photograph to which shot noise was artificially added. **D**. The PSNR was compared between the original image and the Okada- or median-filtered images shown in **C**. *P = 1.03×10^−20^ and 2.44×10^−21^, respectively, *t*_15_ = 32.5, 34.6, paired *t*-test (*F*_15,15_ = 1.43, 1.08, *F*-test of equality of variances), mean ± SEM of 16 compartments.

One representative video frame of fMCI is shown in Fig 13A, which contains shot noise because it was taken under low-light intensity conditions. Because the data were presented in the form of a 16-bit digital image, α was set to 10^6^. When the Okada filter and the median filter were applied to this image, shot noise was reduced and the contours of individual neurons could be more easily identified (Fig 13A). We also filtered a natural photograph (portrait) in which the shot noise was artificially added. The Okada filter and the median filter both successfully smoothed out shot noise (Fig 13C). In both images, we calculated the PSNR to evaluate the denoising effect of the Okada and median filters. The PSNR of the fMCI image in Fig 13A and the photograph in Fig 13C were both higher than the original image (Fig 13B, original *versus* Okada, original *versus* median, *P* = 1.8×10^−5^, *Z* = 4.29, Wilcoxon signed rank test, *F*_23,23_ = 2.13, 2.20, *F*-test of equality of variances, *n* = 24 subregions; Fig 13D, *P* = 1.03×10^−20^ and 2.44×10^−21^, *t*_15_ = 32.5 and 34.6, paired *t*-test, *F*_15,15_ = 1.43 and 1.08, respectively, *n* = 16 compartments.). In the fMCI image, the Okada filter was slightly inferior to the medial filter in terms of the PSNR evaluation (*P* = 4.5×10^−12^, *t*_23_ = 13.0, paired *t*-test, *F*_23,23_ = 1.03).

## Discussion

In this study, we devised an Okada filter, a novel nonlinear filter that is optimized to data with low S/N and with low sampling rates. Indeed, the Okada filter reduces noise in fMCI data and improves their S/N. We compared the performance of the Okada filter to that of the median filter, binomial filter and Savitzky-Golay filter. The Okada filter surpassed the median filter in denoising and computational speed. Moreover, the Okada filter showed a higher computational speed than the binomial and Savitzky-Golay filters. Therefore, the Okada filter is more suitable for practical use. The frequency response analysis revealed that the Okada filter broadly reduces high frequency components, suggesting that the Okada filter works more effectively for white noise-like background fluctuations, such as thermal noise. In addition, the modified Okada filter also improved the S/N of the fMCI data. One may be able to adjust the parameter β to a given purpose.

The S/N in fMCI has been improved by development of more sensitive calcium indicators and more optimized optical systems, such as objective lens and photodetectors [17]; however, their shot noise is stochastically emitted during the process of photon conversion and can come from cosmic rays. Thus, online removal of the shot noise during image acquisition is technically difficult. Therefore, fMCI data are usually denoised using offline filters that are employed in image processing and sound processing [10,18,19]. Each filter has advantages and disadvantages. Linear filters are usually effective in reducing high-frequency noise, including shot noise; however, because of their nature, they often blur the edges of the signal and thereby deform the shapes of the signal. Conversely, nonlinear filters have less influence on the edges, but their denoising powers may be insufficient. In this way, the Okada filter reduces noise by averaging values within a window, such as the linear filter, and leaves the putative signal, such as the median filter, thereby benefitting from the advantages of both types of filters. Furthermore, we found that the Okada filter with longer windows improved the S/N of fMCI traces more efficiently. Because the amplitudes of signal tended to be more severely reduced when using longer windows (relatively to the frame rates), the Okada filters with long windows may be suitable for fMCI data recorded at higher temporal resolution. In the Okada filter, the parameter β is modifiable to optimize its denoising power depending on the S/N of the original data. Some of the recently developed calcium indicators are sensitive enough to exhibit the signal amplitude larger than 50% over the baseline intensity. In such cases with excellent S/N, higher β values might be suitable.

Another disadvantage of nonlinear filters is, in general, the high cost for von Neumann computation; however, the Okada filter is a nonlinear filter that exhibits more rapid computation than the median filter. Its computational speed is almost a match for that of simple linear filters. This efficient computation may be applicable to an immediate filter that works online during data sampling. Moreover, we packed the data processing of the Okada filter into a single mathematical formula, while the median filter cannot be formulated in a single algebraic equation. The equation of Okada filter is a continuous, integrable, and differentiable function. Thus, it is not only arithmetically tractable but may be also applicable to development of electric circuit-based denoising devices.
